# Verification of mesenchymal stem cell injection therapy for interstitial cystitis in a rat model

**DOI:** 10.1371/journal.pone.0226390

**Published:** 2019-12-12

**Authors:** Jae-Wook Chung, So Young Chun, Eun Hye Lee, Yun-Sok Ha, Jun Nyung Lee, Phil Hyun Song, Eun Sang Yoo, Tae Gyun Kwon, Sung Kwang Chung, Bum Soo Kim

**Affiliations:** 1 Department of Urology, School of Medicine, Kyungpook National University, Kyungpook National University Chilgok Hospital, Daegu, Republic of Korea; 2 BioMedical Research Institute, Joint Institute for Regenerative Medicine, Kyungpook National University Hospital, Daegu, Republic of Korea; 3 Department of Pathology, School of Medicine, Kyungpook National University, Daegu, South Korea; 4 Joint Institute for Regenerative Medicine, Kyungpook National University, Daegu, Republic of Korea; 5 Department of Urology, Yeungnam University College of Medicine, Daegu, Republic of Korea; 6 Department of Urology, School of Medicine, Kyungpook National University, Kyungpook National University Hospital, Daegu, Republic of Korea; Università degli Studi della Campania, ITALY

## Abstract

**Objective:**

Interstitial cystitis (IC) is a chronic intractable disease. Recently, the potential application of stem cell (SC) therapy was suggested for IC management. This study aimed to establish an optimal SC source and verify the efficacy and safety of SC injection therapy in an IC rat model.

**Design:**

After IC animal model induction, urine-derived stem cells (USCs), adipose tissue-derived stem cells (ADSCs), bone marrow-derived stem cells (BMSCs) and amniotic fluid-derived stem cells (AFSCs) were injected into the bladder submucosa. The following parameters were analysed: 1) functional improvement of bladder via cystometry, 2) histological changes and 3) inflammatory gene expression and regenerative potential of damaged bladder tissues. Additionally, an optimal method for SC introduction in terms of effective bladder regeneration was analysed.

**Results:**

Intercontraction interval was significantly increased and inflammatory reactions and fibrotic changes were decreased in all of the SC-injected groups than in the control group. PCR analysis revealed that inflammatory gene expression significantly decreased in the USC-treated group than in the other groups. To confirm the optimal SC injection route in the IC rat model, group was divided according to the following criteria: 1) direction of SC injection into the bladder submucosa, 2) injection via tail vein, 3) transurethral instillation. In each analysis, the groups in which SCs were injected into the bladder submucosa showed significantly longer intercontraction interval, better morphologic regeneration and inhibition of bladder inflammatory reaction compared with the other groups.

**Conclusion:**

Regardless of the cell source, human tissue-derived mesenchymal SCs regenerated damaged bladder tissue, promoted functional recovery and inhibited inflammatory cell accumulation in an IC rat model; particularly, USC had the highest inhibitory effect on inflammation. Additionally, direct USC injection into the bladder submucosa was expected to have the best therapeutic effect, which will be an important factor for clinical applications in the future.

## Introduction

Interstitial cystitis (IC) or bladder pain syndrome (BPS) is a chronic inflammatory bladder disease entity characterised by chronic pelvic pain during bladder distention and urinary symptoms, such as frequency, dysuria, urgency and nocturia without any infection of bacteria or definite identifiable pathology. [1, 2] In the 19th century, it was described for the first time by the presence of reddish, bleeding lesions on the bladder mucosa, known as Hunner’s lesions. [3] In this regard, IC is known as a chronic, non-infectious and potentially inflammatory disease of the urinary bladder. [4] The morbidity of IC/BPS ranges from 1 in 100,000 to 5.1 in 1000 across the population worldwide. [5] Because of these various symptoms, patients suffering from IC typically present sleep and sexual disorders, psychological stress, depression and anxiety. [6] It is known that IC deteriorates the quality of life for 3.3–7.9 million people in the United States alone. [7] The pathophysiology of IC is not yet completely understood. However, the loss and destruction of the glycosaminoglycan (GAG) layer from the superficial urothelium and the presence of toxic urinary substances have been suggested as etiology. [8] Such a poor understanding of IC pathophysiology still make the development of definitive therapeutic modalities and establishment of proper animal models for investigative researches challenging. Several treatment options currently in practice are based on the capability to repair the urothelium by replacing lost proteoglycans. However, these treatment options had limited effectiveness with regard to recurrence. [6] Especially, even though oral medicines used in clinical practice, such as amitriptyline and pentosan polysulphate sodium, or interventional methods such as hydrodistension and intravesical instillation therapy are being widely used at present, to date, no definite standard therapeutic modalities have been established for IC. [9]

Recently, several clinical studies have proposed new potential therapeutic options for IC. [10–13] Mesenchymal stem cell (MSC) therapy has been proposed as a reasonable treatment option for many bladder disorders. [14] SCs have the potency to regenerate damaged cells and tissues by differentiating into target cells and regulating a microenvironment favourable for tissue repair. [15, 16] However, to date, there have only been a few trials that have investigated the efficacy of MSC therapy for treating IC.

Therefore, in vivo experiments were performed to select the optimal MSC source for the treatment of IC and determine an appropriate injection SC route to enhance SC function.

## Materials and methods

### Generation of IC animal model

In 2017, we have already demonstrated that the injection of uroplakin II (UPK) generated the most effective IC animal model, showing consequent urothelial barrier loss, inflammatory reaction, tissue fibrosis stimulation, and persistent hyperactive bladder. [17] Following the same protocol, 25 female Sprague–Dawley rats aged 8 weeks were administered UPK.

All rats were anesthetized by intramuscular injection of 16 mg/kg of xylazine (Rompun^®^) and 0.04 mg/kg of tiletamine and zolazepam hydrochloride (Zoletil^®^). We euthanized rats using CO2 gas or cervical dislocation. We injected Rumpun^®^ and Zoletil^®^ intramuscularly once and added 1/3 of the initial dose if anesthesia is poor. In case of unexpected pain or decreased dietary intake, buprenorphine is administered at concentration of 0.05–0.1mg/kg. Unexpected pain was defined as an increase in more than two points of ‘Orbital Tightening', ‘Nose/Cheek Flattening', ‘Ear Changes’ and ‘Whisker Change’ scores by observing on the rat’s face according to the Rat Grimace Scale. We identified and recorded the clinical symptoms and condition of rat for endpoint setting when the animals continue to lie down due to a movement disorder, show reduced intake of food, and lose more than 20% of the normal weight. Hematologic examination is performed to determine a rat’s endpoint when skin sutures are opened, abdominal distension or ocular turbidity are observed after surgery. In addition, when symptoms such as gross hematuria, decreased urine volume, excessive frequency of urination, and pain during urination occur, we regraded these points as deterioration of excessive bladder damage and humanitarian termination is determined. An autopsy is then performed after termination and ascites and histopathological examinations (e.g., significant cortical lymphocytic necrosis in the thymus, mild lymphocytic atrophy in the spleen, etc.) were performed to figure out the cause of death from the administration substance or other abnormalities.

#### 1. Selection of MSCs based on IC treatment efficiency

**(1) Cell preparation**. For urine-derived stem cell (USC) preparation, a sample of voided urine (100 mL) from a healthy male volunteer was collected. The centrifuged cell pellets were washed with PBS containing 5% Penicillin/Streptomycin (P/S, Sigma-Aldrich), and then resuspended and plated in 12-well plates at a concentration of 1000 cells per well in a mixed medium composed of keratinocyte-serum free medium and progenitor cell medium (Gibco, Carlsbad, CA, USA) in a 1:1 ratio.

For amniotic fluid-derived stem cell (AFSC) preparation, amniotic fluid was collected from routine amniocentesis at a gestational age of 16 weeks. Amniotic fluids (10 mL) were centrifuged, and cell pellets were suspended in Chang Medium (Gibco) containing a-MEM, 15% FBS, 18% Chang B and 2% Chang C (Irvine Scientific, Irvine, CA, USA) in a petri dish.

For adipose tissue-derived stem cell (ADSC) preparation, liposuction and centrifugation were performed using a commercial machine (Lipokit, Medikan, Seoul, Korea). After washing with PBS containing 5% P/S, debris was removed. The sample was digested with 0.075% Collagenase Type I (Sigma-Aldrich) prepared in PBS containing 2% P/S for 30 min at 37°C. After incubation, the sample was neutralised with 5 ml α-MEM containing 20% foetal bovine serum (FBS, Gibco). The sample was pipetted several times to avoid aggregation and centrifuged at 2000 rpm for 5 min to obtain stromal vascular fraction (SVF). The supernatant was aspirated, and the cell pellet was suspended in 3 mL of stromal medium (alpha-MEM, Gibco) containing 20% FBS, 1% L-glutamine (Sigma-Aldrich) and 1% P/S. The cell suspension was plated in a 24-well plate at a concentration of approximately 500 cells/well.

Bone marrow-derived stem cells (BMSCs) were purchased from ATCC (PCS-500-012) and cultured in the recommended medium (PCS-500-030). The cells used in this experiment are owned by our team and have been confirmed to have MSC characteristics through previous reports; USC [18], AFSC [19], and ADSC. [20]The adherent cells were cultured, and less than five passages were used in this study.

**(2) Study design**. There were five experimental groups in this study: USC, AFSC, ADSC, BMSC and PBS (control) (each group, n = 5). One week after IC development, catheterization was performed. Low abdominal incision about 2 cm was made. Next, the bladder was exposed and a tiny incision about 2 mm was made at the bladder dome area. Next, one ends of polyethylene-50 tube was positioned in the bladder dome, and the bladder was approximated water-tightly. The other ends of the tube was passed through the subcutaneous layer of the left flank area and extracted out from the posterior neck area. The polyethylene-50 tube was fixed and each SC was injected into the bladder mucosa using Hamilton syringe and low abdominal wound was closed. An SC concentration of 1×10^5^ cells/rat was injected in four sites of the bladder mucosa (anterior wall, dome, right lateral wall and left lateral wall, and the cell concertation at each site was 2.5×10^4^).

**(3) Cystometry, histological, immunohistochemical (IHC) and gene expression analysis**. Cystometry was performed 3, 7 and 14 days after SC injection. The number of voiding episodes was measured at 5 and 10 days after injection of SCs. Bladder was excised 14 days after injection of SCs for histological and gene expression analysis.

Half of each bladder was fixed in 4% paraformaldehyde on 14 days after IC (5 rats of each group were euthanized). The paraffin-embedded bladder samples were sliced into 5-μm sections for hematoxylin and eosin (H&E), toluidine blue, and Masson’s trichrome staining for histology, mast-cell infiltration, and fibrosis analysis, respectively. For toluidine blue staining, bladder sections were washed with xylene to remove the paraffin, and then the slides were processed with an ethanol series. The slides were stained with toluidine blue for 4 min, processed with an ethanol series, and then mounted. The unit area for measuring of mast cells were mm^2^, and lamina propria area was observed. For Masson’s trichrome staining, de-paraffinized and rehydrated sections were re-fixed in Bouin’s solution, stained in Weigert’s iron hematoxylin working solution / Biebrich scarlet-acid fuchsine solution, and treated in phosphomolybdic-phosphotungstic acid solution until the collagen was red. The sections were transferred to aniline blue solution and treated in 1% acetic acid solution. The collagen fibers were stained as blue color.

For IHC, CD3, CD31, UPK3, Zo-1 antibodies (abcam, Cambridge, UK, 1:200 dilution) were used to detect cytotoxic T cell, endothelial cell, inner membrane and intracellular side of the plasma membrane, respectively. The deparaffinised slides were processed in 0.2% Triton-X 100 for 10 min and incubated with 200 μL of 5% FBS in PBS for 2 h. The primary antibody was treated overnight at 4°C, while the secondary antibody (Alexa Fluor 594 Goat anti-rabbit IgG, abcam, 1:1000 dilution) was incubated for 1 h at room temperature.

The remaining half of each bladder tissue was prepared for real-time polymerase chain reaction (PCR) analysis. Total ribonucleic acid (RNA) was isolated using the RNeasy Mini Kit (QIAGEN, Valencia, CA, USA) and complementary deoxyribonucleic acid (cDNA) was prepared using Reverse-Transcription Reagents (Applied Biosystems, Carlsbad, CA, USA) according to the manufacturer’s instructions. PCR was carried out in a real-time PCR machine and analyzed using 7300 System SDS Software (Applied Biosystems). The conditions for the PCR using SYBR^®^ Green PCR Master Mix (Bio-Rad, Hercules, CA, USA) were 95°C for 10 min, followed by 45 cycles of 95°C for 10 sec, 58°C for 50 sec, and 72°C for 20 sec. To verify the relative changes in gene expression, the Ct value for the target gene was normalized to its endogenous control and transformed to a relative gene expression value using the 2^−ΔΔCt^ method. The target genes were MPO, IL-1β, IL-6, IL-17α, TLR4, TLR5 and TLR11.

Especially, IHC with CD3 and toluidine blue staining were for cytotoxic T cell and mast cell detection. Also, H&E stained slides were read and described by a pathologist in this study about the appearance of neutrophils, basophils, monocytes (monocytes, macrophages, lymphocytes, plasma cells) and fibroblasts involved in acute and chronic inflammatory reactions.

#### 2. Optimisation of MSC injection route for IC treatment

**(1) Study design**. The groups were divided into three, including direct MSC injection into the bladder submucosa, instillation of MSC via the urethra and injection of MSC via the tail vein (each group, n = 5).

IC was induced in the rats of each group. One week after IC development, catheterization for cystometry was performed according the same methods described above, and USC (1×10^5^ cells/rat) was injected into the bladder submucosa directly using Hamilton syringe, instilled transurethrally or injected via the tail vein.

(2) Cystometry, histological, immunohistochemical (IHC) and gene expression analysis

Cystometry was performed at 3, 7 and 14 days after SC injection. The number of voiding episodes was measured at 5 and 10 days after SC injection. The bladder was excised 14 days after SC injection and histology, IHC, toluidine blue and Masson’s trichrome staining were also performed. Real-time PCR conditions were the same as described in the methods above, and the target genes were MPO, IL-1β and TNFα.

#### 3. Statistical analysis

All raw data are presented as the means ± standard deviations. Differences in cystometric results, mast cell count and real-time quantitative PCR outcomes were verified by Student’s *t*-test and one-way analysis of variance (ANOVA). When the value was found to be significant by ANOVA, Tukey’s *post-hoc* test was performed. Statistical analysis was performed using SPSS 16.0 for Windows (SPSS Inc., Chicago, IL, USA), and a p value of <0.05 was considered to be statistically significant.

#### 4. Ethics statement

All animal study protocols were approved by the institutional animal ethics committee of Yeungnam University College of Medicine (YUMC-2015-028). The Ethics Committee of Kyungpook National University Chilgok Hospital provided approval for obtaining human samples (KNUCH 2018-08-008-003), and all patients gave their informed consent before providing their samples.

## Results

### 1. Selection of MSCs based on IC treatment efficiency

#### (1) Cystometry

All rats were treated with UPK subcutaneously, and each SC was subsequently injected. The mean intercontraction intervals of each cell treatment group were measured by conscious cystometry. The mean intercontraction intervals of PBS-, USC-, AFSC-, ADSC- and BMSC-injected groups on day 3 were 48.0±14.8, 115.0±40.9, 116.7±5.8, 116.7±25.2 and 158.7±20.1 s; on day 7, they were 81.7±46.5, 124.3±25.2, 123.3±32.1, 186.3±24.7 and 133.3±45.5 s and on day 14, they were 71.3±18.5, 179.3±54.4, 250.0±17.3, 210.0±79.4, 193.7±66.7 s, respectively. All SC-treated groups showed significantly longer mean intercontraction interval compared to control (PBS) group, but the difference among the SC groups was non-significant (p>0.05). Additionally, voiding times per hour of all SC-treated groups were also significantly less than those of control group on days 5 and 10 (Table 1).

**Table 1:** Comparison of voiding number per hour among USC-, AFSC-, ADSC-, BMSC- and PBS-injected groups.

	USC	AFSC	ADSC	BMSC	PBS	*p* value
Day 5	3.0±2.2	3.0±2.9	5.9±4.7	5.7±3.9	15.3±2.9	>0.05
Day 10	2.6±1.9	3.1±2.5	5.4±4.8	4.6±3.2	16.3±6.1	>0.05

#### (2) Histology and gene expression

**Fig 1:**
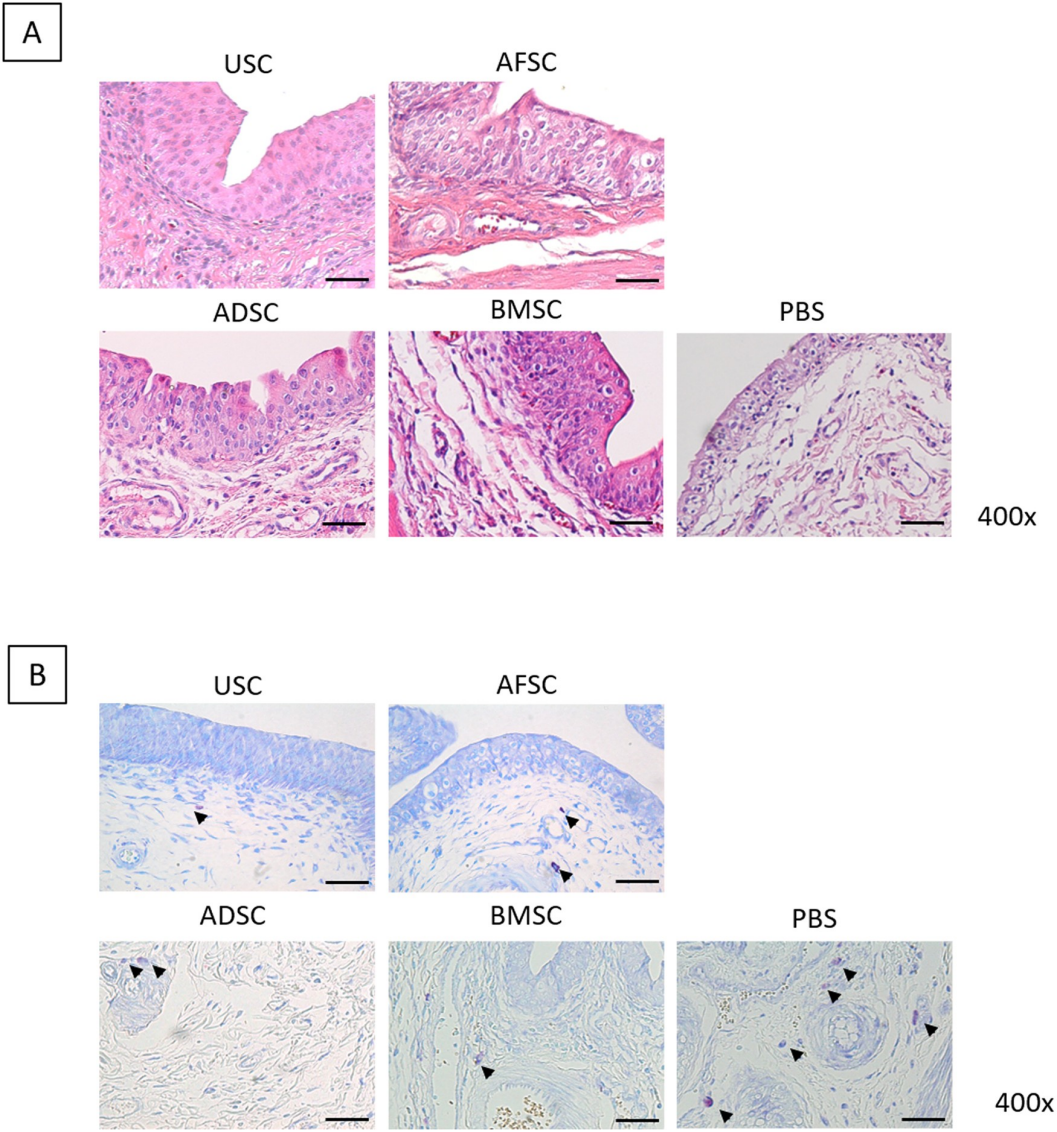
Histological results in USC-, AFSC-, ADSC-, BMSC- and PBS-injected groups. (A) H&E histological examination showed no significant differences in urothelium and smooth muscle regeneration among stem cell (SC)-treated groups. (B) SC-injected groups showed significantly reduced positive mast cell numbers compared with PBS group.

Histological analysis by H&E staining (Fig 1A) revealed that the SC injected bladders had regenerated transitional epithelium (3–5 layers), an intact basement membrane, a compact lamina propria, thick smooth muscle bundles such a regular tissues and rare inflammatory cells infiltration. There were no significant histological differences in urothelium and smooth muscle regeneration among SC-treated groups. Whereas the PBS group, as a negative control, revealed insufficient and thin epithelial layers, loose connective tissue, weak smooth muscle bundles and intraepithelial inflammation showing frequent neutrophils, basophils, monocytes (monocytes, macrophages, lymphocytes, plasma cells) and fibroblasts appearance.

**Fig 2:**
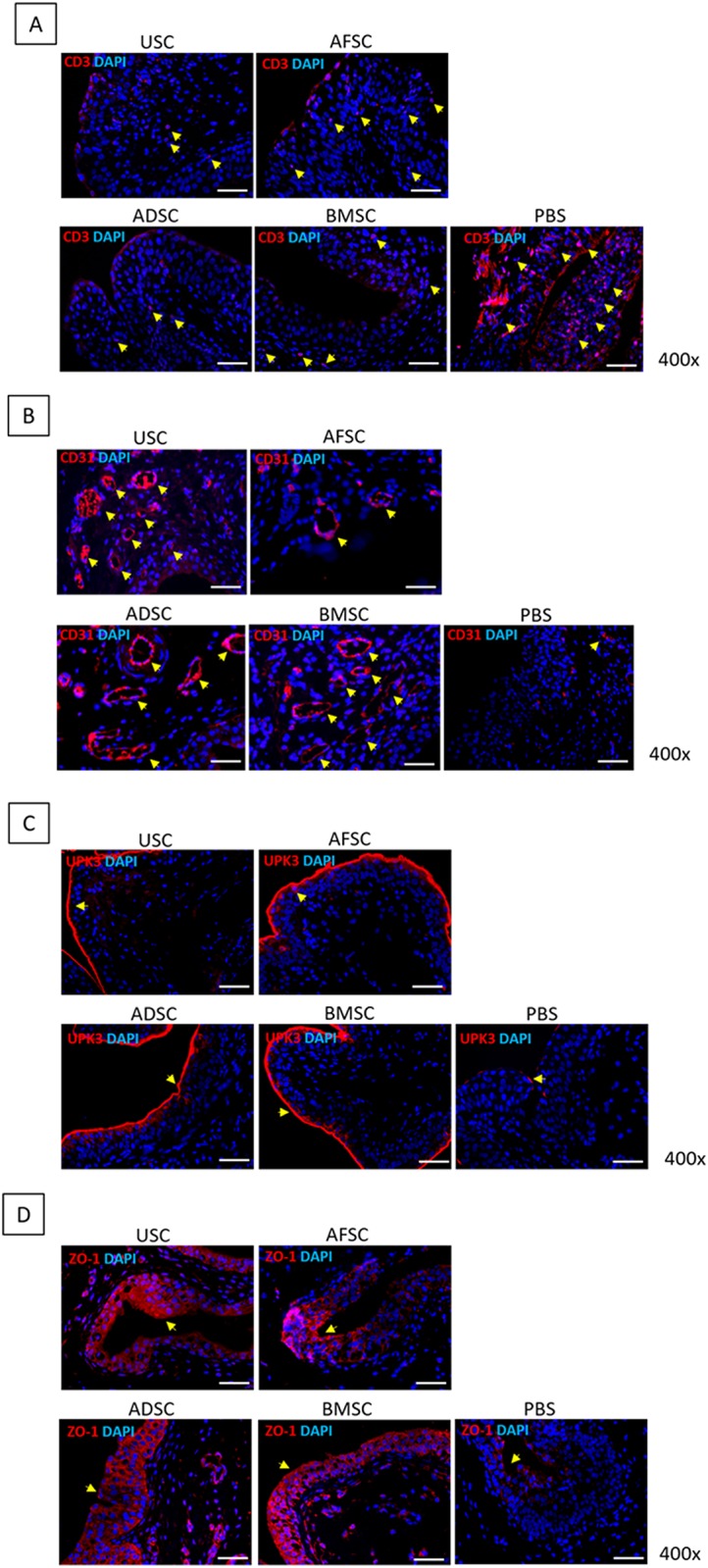
Gene expression results in USC-, AFSC-, ADSC-, BMSC- and PBS-injected groups. (A) CD3-positive T-cells were significantly reduced in SC-treated groups compared with PBS group. (B) CD31-positive cells were higher in USC- and BMSC-injected groups than in AFSC- and ADSC-injected groups. (C) UPK3-positive cell expression was similar among SC-treated groups. (D) ZO-1-positive cell expression was similar among SC-treated groups.

Inflammatory infiltration of mast cells was verified by toluidine blue staining (Fig 1B). In the PBS group, mast cells were frequently revealed in the lamina propria, with a mean count of 31.0±8.0/mm^2^. SC-injected groups showed significantly reduced positive cell numbers compared with the PBS group; the count of mast cells in the USC, AFSC, ADSC and BMSC groups were 21.5±5.19, 13.0±7.0, 21.0±9.6 and 9.0±2.83/mm^2^, respectively. To ensure the inhibition of inflammatory response by SCs, IHC analysis was performed using CD3 antibody for detecting T-cells (Fig 2A). CD3-positive T-cells were significantly decreased in SC-treated groups compared with the PBS group (arrow).

**Fig 3:**
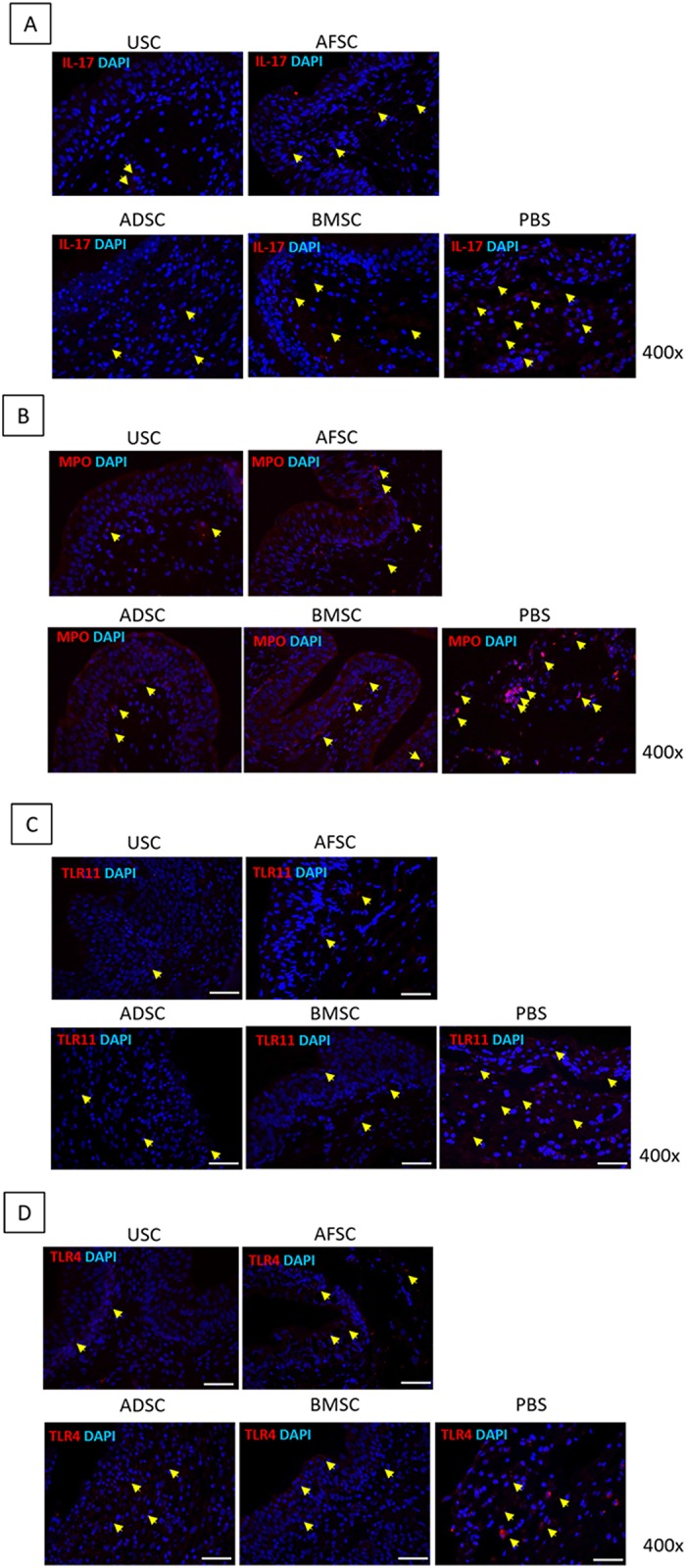
Immunohistochemistry results in USC-, AFSC-, ADSC-, BMSC- and PBS-injected groups. USC-injected group showed significantly inhibited expression of IL-17, MPO, TLR11 and TLR4.

Bladder tissue regeneration was identified using CD31, UPK3 and ZO-1 antibodies for detecting endothelial cells, inner membrane and intracellular side of the plasma membrane, respectively (Fig 2B, 2C and 2D). CD31-positive cells were observed around blood vessels, and their prevalence was higher in the USC- and BMSC-treated groups than in the AFSC- and ADSC-treated groups. UPK3-positive cells were detected at the border and inner layer of the urothelium, and the thickness was very similar for all SC-treated groups. ZO-1 positive cells were revealed in the intracellular region of the urothelial layer, and the thickness of the expression layer was similar for all SC treated groups. Additional IHC analysis revealed that IL-17, MPO, TLR11 and TLR4-positive cells were the lowest in USC injected group (Fig 3A, 3B, 3C and 3D).

**Fig 4:**
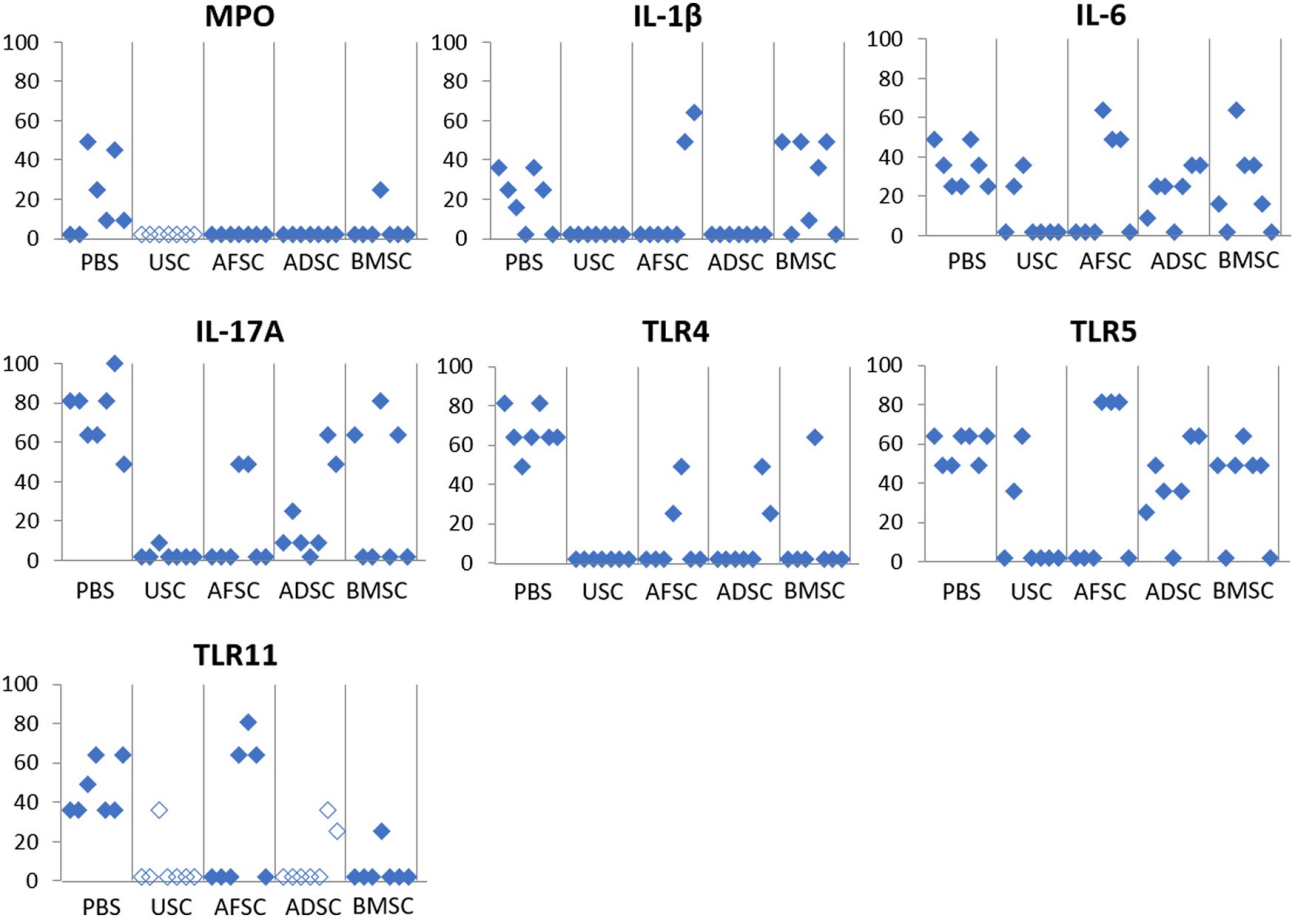
Gene expression results in USC-, AFSC-, ADSC-, BMSC- and PBS-injected groups. USC-injected group showed significantly inhibited expression of MPO, IL-1β, IL-6, IL-17α, TLR4, TLR5 and TLR11.

For gene level inflammatory reactions analysis, the expression of pro-inflammatory genes was verified (Fig 4). Expression of MPO, IL-1β, IL-6, IL-17α, TLR4, TLR5 and TLR11 genes was significantly lower in the SC-treated groups than in the PBS group. Especially, the USC injected group showed significantly inhibited expression of these genes. Therefore, USC was selected as an appropriate SC source for IC treatment.

### 2. Optimisation of USC injection route for IC treatment

#### (1) Cystometry

To optimise the USC injection route, a three-way administration was implemented: direct injection of USC into the bladder submucosa (Group 1), transurethral instillation of USC (Group 2) and USC injection via the tail vein (Group 3). The mean intercontraction intervals of the Groups 1, 2 and 3 on day 3 were 151.7±21.0, 118.6±18.6 and 115.0±7.0 s; on day 7, they were 157.0±26.5, 111.7±37.4 and 142.0±13.1 s; and on day 14, they were 149.3±19.1, 78.3±18.6, 139.3±54.4 s, respectively. Group 1 showed a consistently longer mean intercontraction interval compared with the other groups during the entire experimental period.

Voiding times per hour are shown in Table 2. There were no significant differences in the voiding times among the groups on days 5 and 10.

**Table 2:** Comparison of voiding number per hour among the following groups: Group 1, direct injection of USC to the bladder submucosa; Group 2, transurethral instillation of USC; Group 3, injection of USC via the tail vein.

	Group 1	Group 2	Group 3	*p* value
On day 5	14.6±16.2	13.2±16.4	10.0±8.7	>0.05
On day 10	8.7±7.4	8.3 ±7.2	10.0±0.0	>0.05

#### (2) Histology and gene expression

**Fig 5:**
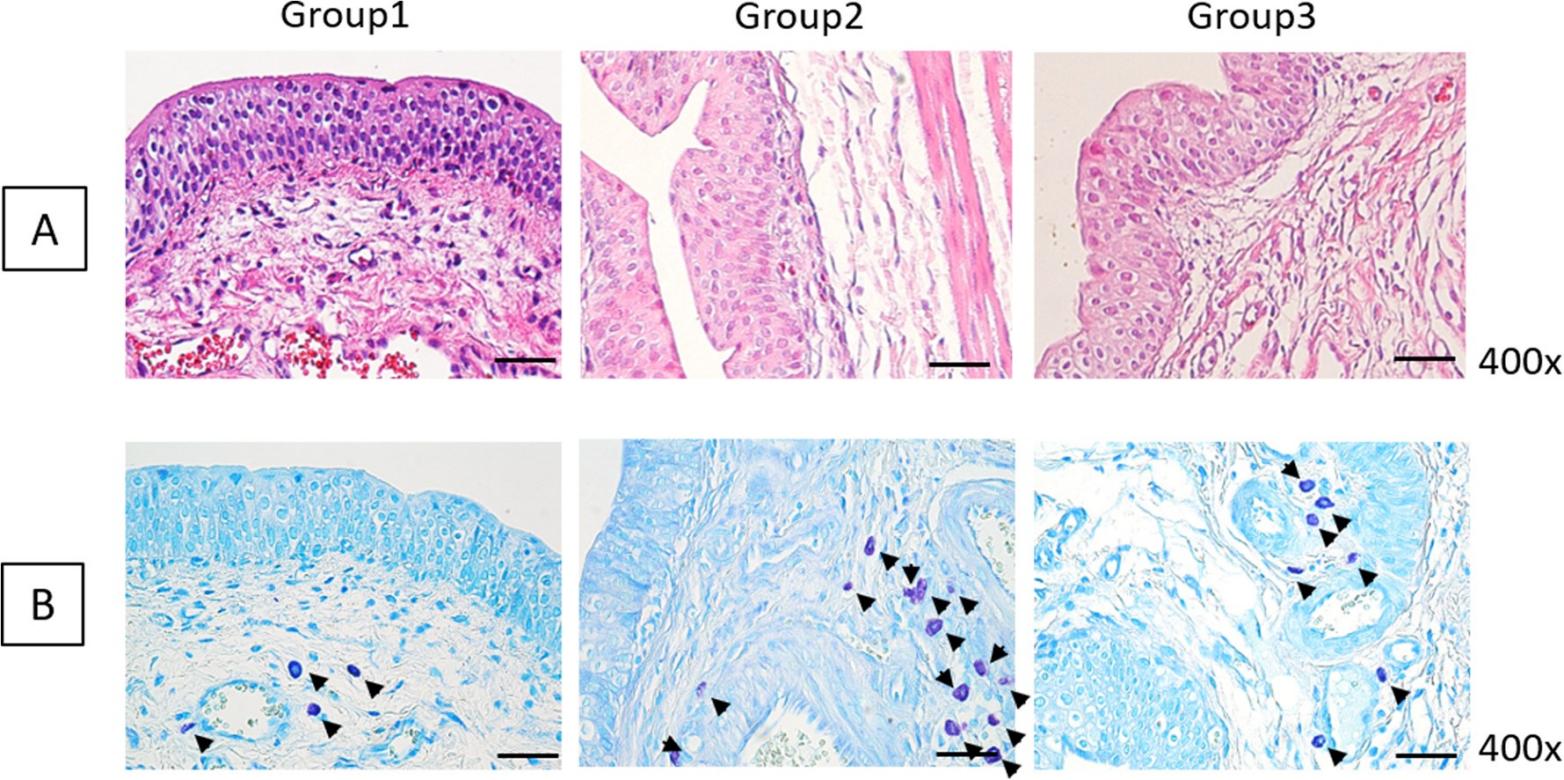
Histological results of direct USC injection into the bladder submucosa (Group 1), transurethral instillation of USC (Group 2) and USC injection via the tail vein (Group 3). (A) H&E histological examination revealed relatively well-regenerated bladder tissues in Group 1 than in other groups. (B) Inflammatory mast cell infiltration was significantly diminished in Group 1.

In H&E histological examination (Fig 5A), direct USC injection into the submucosal layer of the bladder showed a relatively well-regenerated bladder tissue, such as 3–5 layers of transitional epithelium, intact basement membrane, compact lamina propria, thick smooth muscle bundles and rare inflammatory cell infiltration.

**Fig 6:**
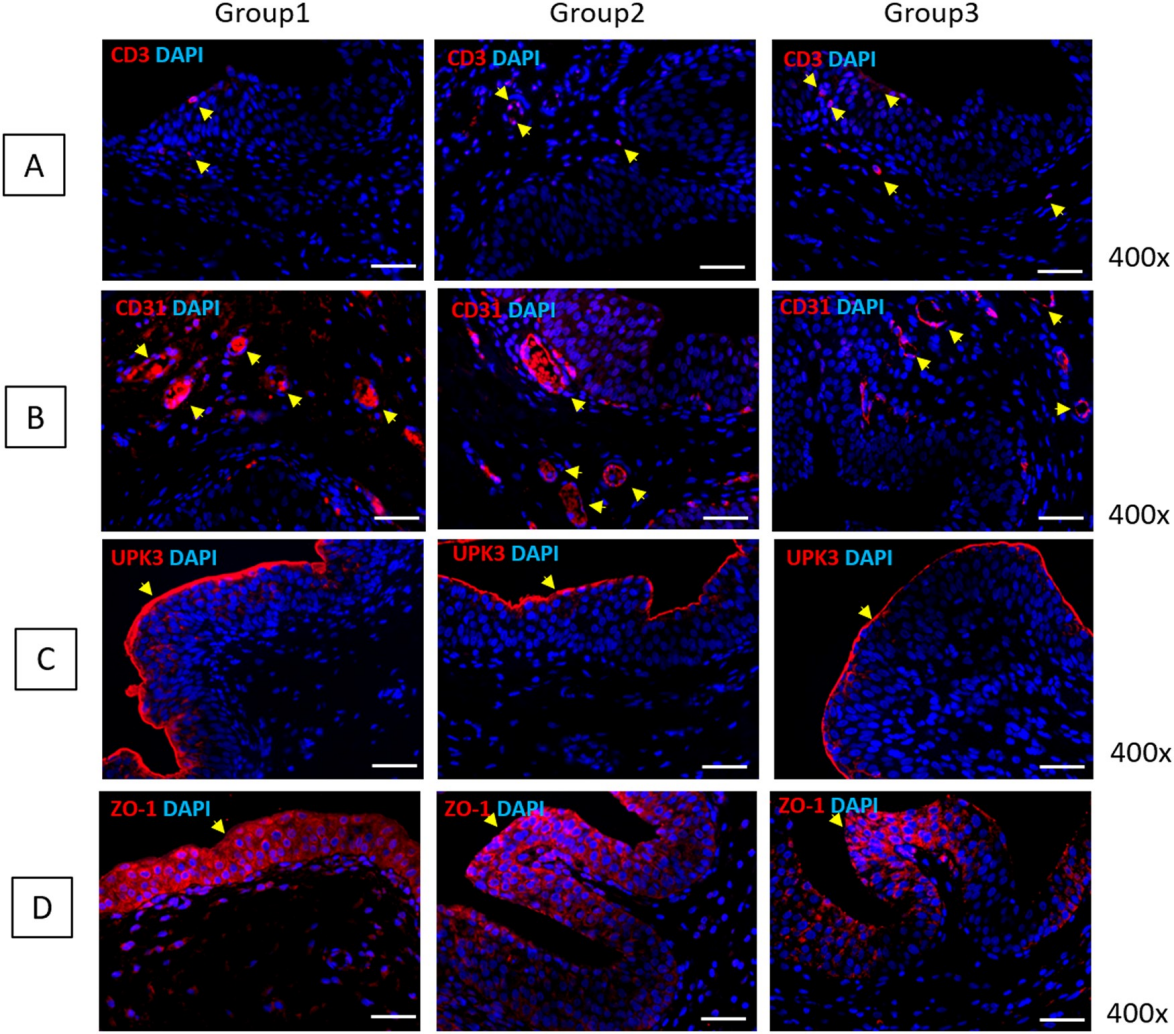
Gene expression results of direct USC injection into the bladder submucosa (Group 1), transurethral instillation of USC (Group 2) and USC injection via the tail vein (Group 3). (A) Group 1 showed significantly reduced CD3-positive cells compared with other groups. (B, C, D) In Group 1, CD31-positive cells were more frequently expressed around blood vessels and the UPK3-positive area was thicker and ZO-1 expression was wider than those in Groups 2 and 3.

Additionally, inflammatory mast cell infiltration was significantly diminished in the bladder submucosa injection group at a mean count of 8.5±3.53/mm^2^ (Fig 5B). In the transurethral instillation group, mast cells were frequently present at a mean count of 30.67±3.78/mm^2^. The intravenous injection group revealed lower prevalence of mast cells (21.33±11.01/mm^2^). This phenomenon was confirmed via IHC analysis using CD3 antibody (Fig 6A). The bladder submucosa injection group showed a significantly reduced concentration of CD3-positive cells compared with that in the other groups (arrows).

Subsequently, bladder tissue regeneration was identified via IHC staining using CD31, UPK3 and ZO-1 antibodies (Fig 6B, 6C and 6D). In the bladder submucosa injection group, CD31-positive cells were more frequently expressed around blood vessels, UPK3-positive area was thicker and ZO-1 expression was wider than the transurethral instillation and intravenous injection groups.

**Fig 7:**
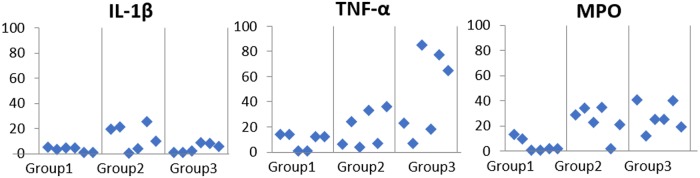
Gene expression results of direct USC injection into the bladder submucosa (Group 1), transurethral instillation of USC (Group 2) and USC injection via the tail vein (Group 3). IL-1β, TNF-α and MPO gene expression was significantly lower in Group 1.

Pro-inflammatory gene expression analysis revealed similar results for toluidine blue and IHC staining (Fig 7). The expression of IL-1β, TNF-α and MPO genes were significantly lower in the bladder submucosa injection group.

## Discussion

The pathophysiology of IC is complicated and is challenging to understand. One of the possible candidate pathogenesis for IC is an increased mast cells count in the bladder mucosa. [21] It is a well-known fact that an allergic reaction activates mast cells. Cytokine such as vasoactive intestinal peptide and tumour necrosis factor secreted by these allergic reaction play important roles in the IC pathophysiology. [22] The autoimmune mechanism of IC is another important pathophysiology suggested over the recent years based on studies of an association between autoimmune diseases and IC. [23] The histology of IC patient’s bladder are composed of infiltration with mast cells, T cells, monocytes and plasma cells. [24] However, the mechanisms underlying the relationship between IC and autoimmune diseases still remain unclear. This complex nature makes it difficult to develop a definite treatment and to establish a standard animal model for translational research.

SCs have self-renewal ability for maintaining their own population pool and have the potential to simultaneous develop differentiated progenitors for regenerating the cells that are used up during the lifetime of an individual or damaged by tissue injuries. [25] Therefore, SCs play an important role in maintaining tissue and organ integrity of an adult body. As research on SCs has broadened the perspective of researchers and clinicians on several intractable disorders, SC therapy is being considered as a new treatment modality for various intractable bladder disease. [26] Several preclinical or clinical trials have revealed that the transplanted SCs directly replace the damaged tissue cor give rise to therapeutic outcomes via the action of paracrine factors, such as growth factors and cytokines released by transplanted SCs. [27]

MSCs have the ability to interact with many kinds of immune cells, including B cells, T cells, dendritic cells (DCs), natural killer (NK) cells, neutrophil, and macrophages. Mechanisms of interaction were shown to rely on cell–cell contact working in collaboration with secretion of soluble immune factors to induce MSC-regulated immunosuppression. These specific modulators, including a multitude of immune-modulatory factors, cytokines, and growth factors, modulate inflammatory responses and balance immune profiles. Namely, soluble immune secretomes, such as prostaglandin E2 (PGE-2), indoleamine 2,3-dioxygenase (IDO), or nitric oxide (NO), respond to immune cells to activate immunoregulation by MSCs. [28] The cells used in this experiment are reported to have these immune-modulatory factors, thus we expect little immune rejection of the injected human SCs even in the rat model.

Considering these characteristics of SCs, several types of MSCs were administered in this IC animal model to verify the therapeutic effect of SCs after establishing an optimal animal model. As described above, IC is characterised by the loss of integrity of the urothelium; thus, urothelial regeneration by SCs can be an indicator to improve the functional and pathophysiological aspects of IC. A previous study conducted by Adamovicz et al. showed that the conditioned medium (CM) derived from MSC culture can be used for intravesical treatment of IC. [29] CM, which is harvested during the MSC cultivation, contains paracrine factors having anti-inflammatory, anti-apoptotic, anti-fibrotic and immunomodulative properties. Thus, CM can be effective to generate a microenvironment favourable for regenerating of an injured bladder mucosa. More recently, using an orthodox IC animal model, Kim et al. provided experimental evidences that human umbilical cord-blood derived stem cells (UCB-SCs) can provide a stable therapeutic result for IC treatment by regenerating the urothelium via epidermal growth factor (EGF) stimulation and Wnt signalling. [6] They showed that single administration of UCB-SCs into the bladder submucosal layer improved the voiding frequency of IC rats significantly. Histological analysis also revealed that SC therapy cures most pathologic characteristics of IC, including denudation of urothelium and mast cell infiltration, neural network and angiogenesis in the injured bladder mucosa. Based on this trial, USC, AFSC, ADSC and BMSC were evaluated as SC sources in this study; these MSCs have self-renewal, multilineage differentiation capacity [30] and immunomodulatory properties. [31] Histological examination revealed that all kinds of MSCs showed a very similar urothelium and smooth muscle regeneration ability. With regard to the observation of inflammatory mast cell infiltration, MSC-treated groups showed a significantly reduced positive cell number compared than the PBS-treated group. As per IHC analysis, CD3-positive T-cells were also significantly reduced in MSC-treated groups. These anti-inflammatory reactions stimulate bladder tissue regeneration via an immunomodulatory effect. The mechanisms of human MSC immunomodulation are interactions with specific leukocyte populations. [32] MSCs inhibit CD4 T lymphocytes secreting prostaglandin E2 (PGE2) and transforming growth factor β1 (TGF-β1). [33] MSCs modulate T lymphocyte fate from a T helper cell type 1 (Th1) to a Th2 milieu; thus, tumor necrosis factor-α (TNF-α) and interferon-γ (IFN-γ) secretion was inhibited. [34] MSCs suppress cytotoxic T cells activity [35], natural killer cells (NK) [36], B cell maturation [37], myeloid cell production [38] and granulocyte production. [39] The immunomodulatory ability of each MSC was classified in detail with inflammatory gene expression. The pro-inflammatory genes, including MPO, IL-1β, IL-17α, IL-6, TLR4, TLR5 and TLR11, were significantly lower in the USC-injected group than in the AFSC, ADSC and BMSC. Although all of the used MSCs were satisfied for IC treatment based on gene analysis, USC was considered as an appropriate SC source; it was also easy to use USCs in clinical applications as an autologous cell source and could be unlimitedly obtained in a non-invasive manner.

After the selection of an optimal SC source, the cell administration route was evaluated. Regeneration potency and plasticity of MSCs have been well demonstrated under in vitro differentiation conditions. However, low survival and viability or poor engraftment of the transplanted MSCs under in vivo conditions have aroused sceptical aspects about MSC therapy. In fact, a majority of MSC-mediated therapeutic effects have been explained by paracrine factors with anti-inflammatory, cytoprotective, pro-angiogenic and immuno-modulatory effects rather than by direct repair of damaged cells. [40] Understanding the crucial mechanisms of MSC therapy and establishing an efficacious gold standard approach to treat IC is also important. In this trial, depending on the administration route, the fate of MSCs, life span, paracrine secretion and/or immunomodulatory properties could be changed. [41] Thus, to optimise USC therapy with maximum efficacy, three delivery routes were compared: direct injection into the submucosal layer of the bladder, transurethral instillation or intravenous injection via the tail vein. The therapeutic effects of USCs were focused on the promotion of histological tissue regeneration, inhibition of inflammatory reactions and functional recovery. Among the routes, direct injection into the bladder submucosa showed significantly enhanced bladder tissue regeneration and reduced inflammatory reactions in comparison with the transurethral or intravenous route. Each of the routes has its own advantages and disadvantages. Transurethral instillation can be performed in the most minimal-invasive manner and can facilitate adhesion and transmigration of MSCs into the injured urothelium, possibly expanding the treated area. However, the survival rate and biological activity of cells may be reduced because cells are exposed to inappropriate conditions for a prolonged period; moreover, urine is acidic, about 6.0 and composing salts and waste products. In case of an intravenous cell delivery route, physiological homing signal allows the cells to migrate towards the injured tissue with chemotactic response. [42] However, a lesser number of infused cells settle in the injured region because cells are delivered through systemic circulation, during which they are easily trapped into the lungs (emboli) and/or are eliminated by the spleen. [43] Even though, all of the three routes are known to be convenient, minimally invasive and can be used to administer injections multiple times, direct injection into the target tissue can be considered as the most effective route for IC treatment. Although direct injection of SCs into the bladder submucosa was performed via laparotomy in an animal model, it can be performed cystoscopically in case of humans by adopting a less-invasive approach. Therefore, direct injection of SCs into the bladder submucosa was considered as the optimal cell administration route.

For further studies, it is necessary to evaluate the therapeutic effects of SC on bladder pain, which is one of the most bothersome symptoms of IC. Although uroplakin2 treatment produces characteristics similar to the human IC phenotype, including overactive bladder function, persistence of symptoms and histologic alterations of the bladder, it did not cause bladder pain, while uropakin3a can induce all predominant IC phenotypic characteristics, including pelvic pain. Because this study aimed to verify the mid-term regenerative effect (2 weeks) of MSCs, it was considered that persistent chronic pain could cause negative effects on the experimental animals. Thus, we used uroplakin2 to induce an IC animal model in this study. However, we plan to induce another IC animal model with painful bladder and to evaluate the efficacy of SC on bladder pain even with short-term experiment in the next research. Additionally, further longer-term follow-up is warranted. After injection of SCs, 6 months of observation may be required to confirm in vivo safety as well as functional and histological changes in the bladder. The long-term follow-up results also can help determine whether booster injection or cell dose modification is required.

## Conclusions

This study helped identify the optimal MSCs with the highest therapeutic efficacy as well as helped decide the ideal route for SC administration. USCs were an appropriate SC source for improving IC symptoms via inflammatory pathway inhibition. Direct cell injection into the bladder submucosa was the most effective route with significantly enhanced bladder tissue regeneration and reduced inflammatory reactions. These findings might facilitate better pathological understanding of IC as well as development of effective therapeutic strategies for IC treatment.

## Supporting information

S1 Table Raw data (cystometry and RT-PCR). (XLSX)

## Abbreviations

AA: acetic acid
CYP: cyclophosphamide
HCl: hydrogen chloride
IC: interstitial cystitis
IFNγ: interferon γ
IL: interleukin
LPS: lipopolysaccharide
MCP: monocyte chemotactic protein
MPO: myeloperoxidase
PBS: phosphate-buffered saline
PCR: polymerase chain reaction
TLR: Toll-like receptor
UPK: uroplakin II

## References

[1] Bogart LM, Berry SH, Clemens JQ. Symptoms of interstitial cystitis, painful bladder syndrome and similar diseases in women: a systematic review. J Urol. 2007;177(2):450–6. 10.1016/j.juro.2006.09.032 . 17222607

[2] Chancellor MB, Yoshimura N. Treatment of interstitial cystitis. Urology. 2004;63(3 Suppl 1):85–92. 10.1016/j.urology.2003.10.034 . 15013658

[3] Berry SH, Elliott MN, Suttorp M, Bogart LM, Stoto MA, Eggers P, et al Prevalence of symptoms of bladder pain syndrome/interstitial cystitis among adult females in the United States. J Urol. 2011;186(2):540–4. 10.1016/j.juro.2011.03.132 . 21683389 PMC3513327

[4] Phatak S, Foster HE Jr. The management of interstitial cystitis: an update. Nat Clin Pract Urol. 2006;3(1):45–53. 10.1038/ncpuro0385 . 16474494

[5] Hanno P, Keay S, Moldwin R, Van Ophoven A. International Consultation on IC—Rome, September 2004/Forging an International Consensus: progress in painful bladder syndrome/interstitial cystitis. Report and abstracts. Int Urogynecol J Pelvic Floor Dysfunct. 2005;16 Suppl 1:S2–S34. 10.1007/s00192-005-1301-x . 15883858

[6] Kim A, Shin DM, Choo MS. Stem Cell Therapy for Interstitial Cystitis/Bladder Pain Syndrome. Curr Urol Rep. 2016;17(1):1 10.1007/s11934-015-0563-1 . 26686192

[7] Nordling J. Interstitial cystitis: how should we diagnose it and treat it in 2004? Curr Opin Urol. 2004;14(6):323–7. 10.1097/00042307-200411000-00005 15626873

[8] Sand PK. Proposed pathogenesis of painful bladder syndrome/interstitial cystitis. J Reprod Med. 2006;51(3 Suppl):234–40. . 16676918

[9] Kim A, Hoe KO, Shin JH, Choo MS. Evaluation of the incidence and risk factors associated with persistent frequency in interstitial cystitis/bladder pain syndrome and the efficacy of antimuscarinic treatment. Investig Clin Urol. 2017;58(5):353–8. 10.4111/icu.2017.58.5.353 . 28868507 PMC5577332

[10] Adamowicz J, Pokrywczynska M, Drewa T. Conditioned medium derived from mesenchymal stem cells culture as a intravesical therapy for cystitis interstitials. Med Hypotheses. 2014;82(6):670–3. Epub 2014/04/01. 10.1016/j.mehy.2014.02.027 . 24679668

[11] Song M, Lim J, Yu HY, Park J, Chun JY, Jeong J, et al Mesenchymal Stem Cell Therapy Alleviates Interstitial Cystitis by Activating Wnt Signaling Pathway. Stem Cells Dev. 2015;24(14):1648–57. 10.1089/scd.2014.0459 . 25745847 PMC4499842

[12] Ryu CM, Yu HY, Lee HY, Shin JH, Lee S, Ju H, et al Longitudinal intravital imaging of transplanted mesenchymal stem cells elucidates their functional integration and therapeutic potency in an animal model of interstitial cystitis/bladder pain syndrome. Theranostics. 2018;8(20):5610–24. 10.7150/thno.27559 30555567 PMC6276303

[13] El-Hamamsy D. Bladder wall injection of mesenchymal stem cells ameliorates bladder inflammation, overactivity and nociception in a chemically induced interstitial cystitis-like rat model. Int Urogynecol J. 2018 10.1007/s00192-018-3631-5 . 29600399

[14] Lin CS. Stem Cell Therapy for the Bladder-Where Do We Stand? J Urology. 2011;185(3):779–80. 10.1016/j.juro.2010.12.014 21239018 PMC3160447

[15] Maumus M, Guerit D, Toupet K, Jorgensen C, Noel D. Mesenchymal stem cell-based therapies in regenerative medicine: applications in rheumatology. Stem Cell Res Ther. 2011;2(2):14 10.1186/scrt55 . 21457518 PMC3226285

[16] Wang D, Li J, Zhang Y, Zhang M, Chen J, Li X, et al Umbilical cord mesenchymal stem cell transplantation in active and refractory systemic lupus erythematosus: a multicenter clinical study. Arthritis Res Ther. 2014;16(2):R79 10.1186/ar4520 . 24661633 PMC4060570

[17] Song PH, Chun SY, Chung JW, Kim YY, Lee HJ, Lee JN, et al Comparison of 5 Different Rat Models to Establish a Standard Animal Model for Research Into Interstitial Cystitis. International Neurourology Journal. 2017;21(3):163–70. 10.5213/inj.1734898.449 28954463 PMC5636959

[18] Chun SY, Kim HT, Lee JS, Kim MJ, Kim BS, Kim BW, et al Characterization of urine-derived cells from upper urinary tract in patients with bladder cancer. Urology. 2012;79(5):1186 e1–7. 10.1016/j.urology.2011.12.034 . 22381247

[19] Kim BS, Chun SY, Lee JK, Lim HJ, Bae JS, Chung HY, et al Human amniotic fluid stem cell injection therapy for urethral sphincter regeneration in an animal model. BMC Med. 2012;10:94 10.1186/1741-7015-10-94 . 22906045 PMC3520761

[20] Lee SY, Lim J, Khang G, Son Y, Choung PH, Kang SS, et al Enhanced ex vivo expansion of human adipose tissue-derived mesenchymal stromal cells by fibroblast growth factor-2 and dexamethasone. Tissue Eng Part A. 2009;15(9):2491–9. 10.1089/ten.tea.2008.0465 . 19292683

[21] Sant GR, Theoharides TC. The role of the mast cell in interstitial cystitis. Urol Clin North Am. 1994;21(1):41–53. . 8284844

[22] Church MK, Lowman MA, Rees PH, Benyon RC. Mast cells, neuropeptides and inflammation. Agents Actions. 1989;27(1–2):8–16. 10.1007/bf02222185 . 2473641

[23] van de Merwe JP. Interstitial cystitis and systemic autoimmune diseases. Nat Clin Pract Urol. 2007;4(9):484–91. 10.1038/ncpuro0874 . 17823601

[24] Johansson SL, Fall M. Clinical features and spectrum of light microscopic changes in interstitial cystitis. J Urol. 1990;143(6):1118–24. 10.1016/s0022-5347(17)40201-1 . 2342171

[25] Ratajczak MZ, Machalinski B, Wojakowski W, Ratajczak J, Kucia M. A hypothesis for an embryonic origin of pluripotent Oct-4(+) stem cells in adult bone marrow and other tissues. Leukemia. 2007;21(5):860–7. 10.1038/sj.leu.2404630 . 17344915

[26] Wezel F, Southgate J, Thomas DF. Regenerative medicine in urology. Bju Int. 2011;108(7):1046–65. 10.1111/j.1464-410X.2011.10206.x . 21895928

[27] Maltais S, Tremblay JP, Perrault LP, Ly HQ. The paracrine effect: pivotal mechanism in cell-based cardiac repair. J Cardiovasc Transl Res. 2010;3(6):652–62. 10.1007/s12265-010-9198-2 . 20559770

[28] Wang M, Yuan Q, Xie L. Mesenchymal Stem Cell-Based Immunomodulation: Properties and Clinical Application. Stem Cells Int. 2018;2018:3057624 10.1155/2018/3057624 . 30013600 PMC6022321

[29] Adamowicz J, Pokrywczynska M, Drewa T. Conditioned medium derived from mesenchymal stem cells culture as a intravesical therapy for cystitis interstitials. Med Hypotheses. 2014;82(6):670–3. 10.1016/j.mehy.2014.02.027 24679668

[30] Korbling M, Estrov Z. Adult stem cells for tissue repair—a new therapeutic concept? N Engl J Med. 2003;349(6):570–82. 10.1056/NEJMra022361 . 12904523

[31] Wang LT, Ting CH, Yen ML, Liu KJ, Sytwu HK, Wu KK, et al Human mesenchymal stem cells (MSCs) for treatment towards immune- and inflammation-mediated diseases: review of current clinical trials. J Biomed Sci. 2016;23(1):76 10.1186/s12929-016-0289-5 . 27809910 PMC5095977

[32] Uccelli A, Moretta L, Pistoia V. Mesenchymal stem cells in health and disease. Nat Rev Immunol. 2008;8(9):726–36. 10.1038/nri2395 . 19172693

[33] Wang L, Zhao Y, Shi S. Interplay between Mesenchymal Stem Cells and Lymphocytes: Implications for Immunotherapy and Tissue Regeneration. J Dent Res. 2012;91(11):1003–10. 10.1177/0022034512460404 22988011 PMC3490280

[34] Duffy MM, Ritter T, Ceredig R, Griffin MD. Mesenchymal stem cell effects on T-cell effector pathways. Stem Cell Res Ther. 2011;2(4):34 10.1186/scrt75 . 21861858 PMC3219065

[35] Li M, Sun X, Kuang X, Liao Y, Li H, Luo D. Mesenchymal stem cells suppress CD8+ T cell-mediated activation by suppressing natural killer group 2, member D protein receptor expression and secretion of prostaglandin E2, indoleamine 2, 3-dioxygenase and transforming growth factor-beta. Clin Exp Immunol. 2014;178(3):516–24. 10.1111/cei.12423 . 25070361 PMC4238878

[36] Chen PM, Yen ML, Liu KJ, Sytwu HK, Yen BL. Immunomodulatory properties of human adult and fetal multipotent mesenchymal stem cells. J Biomed Sci. 2011;18:49 10.1186/1423-0127-18-49 . 21762539 PMC3156728

[37] Corcione A, Benvenuto F, Ferretti E, Giunti D, Cappiello V, Cazzanti F, et al Human mesenchymal stem cells modulate B-cell functions. Blood. 2006;107(1):367–72. 10.1182/blood-2005-07-2657 . 16141348

[38] Jiang XX, Zhang Y, Liu B, Zhang SX, Wu Y, Yu XD, et al Human mesenchymal stem cells inhibit differentiation and function of monocyte-derived dendritic cells. Blood. 2005;105(10):4120–6. 10.1182/blood-2004-02-0586 . 15692068

[39] Cassatella MA, Mosna F, Micheletti A, Lisi V, Tamassia N, Cont C, et al Toll-like receptor-3-activated human mesenchymal stromal cells significantly prolong the survival and function of neutrophils. Stem Cells. 2011;29(6):1001–11. 10.1002/stem.651 . 21563279

[40] Kim N, Cho SG. New strategies for overcoming limitations of mesenchymal stem cell-based immune modulation. Int J Stem Cells. 2015;8(1):54–68. 10.15283/ijsc.2015.8.1.54 . 26019755 PMC4445710

[41] Harding J, Roberts RM, Mirochnitchenko O. Large animal models for stem cell therapy. Stem Cell Res Ther. 2013;4(2):23 10.1186/scrt171 . 23672797 PMC3706788

[42] Sheng CC, Zhou L, Hao J. Current stem cell delivery methods for myocardial repair. Biomed Res Int. 2013;2013:547902 10.1155/2013/547902 . 23509740 PMC3591183

[43] Kurtz A. Mesenchymal stem cell delivery routes and fate. Int J Stem Cells. 2008;1(1):1–7. 10.15283/ijsc.2008.1.1.1 . 24855503 PMC4021770

